# Classifying high-risk versus very high-risk prostate cancer: is it relevant to outcomes of conformal radiotherapy and androgen deprivation?

**DOI:** 10.1186/s13014-016-0743-2

**Published:** 2017-01-06

**Authors:** Akram Saad, Jeffrey Goldstein, Yaacov R. Lawrence, Benjamin Spieler, Raya Leibowitz-Amit, Raanan Berger, Tima Davidson, Damien Urban, Lev Tsang, Dror Alezra, Ilana Weiss, Zvi Symon

**Affiliations:** 1 Departments of Radiation Oncology, Chaim Sheba Medical Center, Tel Aviv University Sackler School of Medicine, Tel Hashomer, 52621 Ramat Gan, Israel; 2 Medical Oncology, Chaim Sheba Medical Center, Tel Aviv University Sackler School of Medicine, Tel-Hashomer, 52621 Israel; 3 Nuclear Medicine, Chaim Sheba Medical Center, Tel Aviv University Sackler School of Medicine, Tel-Hashomer, 52621 Israel

## Abstract

**Objective:**

To evaluate outcomes in prostate cancer patients classified as high-risk (HR) or very high-risk (VHR) who were treated with conformal radiation therapy (CRT) and androgen deprivation therapy (ADT).

**Methods:**

Between 11/2001 and 3/2012, 203 patients with HR disease received CRT to the prostate (78–82 Gy) and pelvic lymph nodes (46–50 Gy) with ADT (6 m-2 years). Median follow-up was 50 months (12 m-142 m). Biochemical failure was defined according to Phoenix definition. Imaging studies were used to identify local, regional or metastatic failure. Four different VHR/HR groupings were formed using the 2014 and revised 2015 NCCN guidelines. Differences were examined using Kaplan Meier (KM) estimates with log rank test and uni- and multivariate Cox regression analysis (MVA).

**Results:**

Failure occurred in 30/203 patients (15%). Median time to failure was 30 m (4 m-76 m). KM estimate of 4 year biochemical disease free survival (b-DFS) for the entire cohort was 87% (95%CI: 82–92%). Four year KM survival estimates for b-DFS, PCSS and OS were comparable for each NCCN subgroup. On univariate analysis, the NCCN subgroups were not predictive of b-DFS at 4 years, however, DMFS was worse for both VHR subgroups (*p* = .03and .01) respectively. Cox univariate analysis was also significant for: PSA ≥40 ng/ml *p* = 0.001; clinical stages T2c *p* = .004, T3b *p* = .02 and > 4 cores with Gleason score 8–10 *p* < .03. On MVA, only PSA ≥ 40 ng/ml was predictive for b-DFS or MFS at 4 years (HR: 3.75 and 3.25, *p* < 0.005).

**Conclusion:**

Patients with HR and VHR disease treated with CRT and ADT had good outcomes. Stratification into HR and VHR sub-groups provided no predictive value. Only PSA ≥40 ng/ml predicted poor outcomes on MVA. Distant failure was dominant and local recurrence rare, suggesting that improved systemic treatment rather than intensification of local therapy is needed.

**Summary:**

Patients with high-risk prostate cancer are most often treated with conformal dose escalated radiation therapy with androgen deprivation. Stratification into high versus very high-risk subgroups using 2014 or revised 2015 National Comprehensive Cancer Network (NCCN) criteria did not impact treatment outcomes. Only Prostate Serum Antigen (PSA) ≥40 ng/ml was predictive of poor prognosis. Distant failure was dominant and local recurrence uncommon which challenges the notion that intensification of local therapy will further improve outcomes in patients with high-risk disease.

## Introduction

Physicians and patients, when asked about therapy for localized prostate cancer often look to the National Comprehensive Cancer Network (NCCN) guidelines to provide guidance for selection between different treatment options [1]. Since patients with high-risk (HR) disease have a heterogeneous prognosis, this group has been further subdivided to separate patients thought to have the worst prognosis into the very high-risk (VHR) category [1].

Radiation therapy (RT) has long been considered the primary treatment modality for patients with HR disease and is the only treatment considered by the National Comprehensive Cancer Network (NCCN) to have sufficient evidence to support a Category 1 treatment recommendation [1]. Despite the NCCN treatment recommendations based on improved outcomes for HR patients treated with high dose conformal radiation therapy (CRT) and androgen deprivation therapy (ADT), there is growing interest in the use of radical prostatectomy (RP) for patients with HR disease [2, 3]. Justifications given for considering surgery are high rates of local and systemic failure associated with the use of RT as well as reported good outcomes associated with the use of surgery [2, 3].

Sundi et al. defined a VHR group with adverse prognostic factors predictive for poor outcome following surgery and suggested the need for multimodal therapy to improve outcomes [4, 5]. In consideration of these findings, the 2014 NCCN guidelines were revised and added the presence of primary Gleason grade 5 or ≥5 cores with Gleason score 8–10 as new criteria for inclusion into the VHR group [1]. While relevant for surgical outcomes, the predictive value of the HR/VHR grouping has not been assessed in patients treated with current CRT techniques [6]. Recently, Narang et al. showed inferior outcomes in the VHR versus HR group in a cohort of patients treated with RT and ADT from 1993 through 2006. However, this retrospective study was limited by use of radiation techniques, treatment volumes, dose, and use of ADT that do not reflect current therapeutic approaches [6].

We reviewed treatment outcomes in a cohort of patients with HR disease treated with high dose CRT and ADT to determine if local recurrence (LR) or metastatic disease was predominant. Patients were stratified according to the original and revised NCCN guidelines for HR/VHR groups. The value of this classification system to provide prognostic guidance and improved treatment recommendations for patients with HR disease was assessed.

## Methods

### Patients

The radiation oncology prostate cancer database of 509 patients entered between November 2001 and March 2012 was reviewed following approval of the hospital ethics committee. Patients meeting NCCN criteria for HR or VHR disease (*n* = 203) who were treated with CRT were identified. Demographic information, clinical stage, PSA, Gleason grade and score, number and percentage of biopsy cores involved with tumor, use of ADT, and early and late treatment toxicity data were extracted from the electronic medical record. Treatment technique, radiation dose, fraction schedule, target volume and use of image guidance were obtained from the treatment planning system.

The characteristics and treatments of these 203 HR patients are listed in Table 1. Median age was 74 years (range 56 years-89 years). Gleason scores were > 7 in 143 patients and ≤7 in 60 patients. Primary Gleason grade 5 and Gleason score 8–10 in ≥ 5 cores occurred in 17 patients and 82 patients respectively. Median PSA was 15.1 ng/ml (range: 1.4 ng/ml– 449 ng/ml). PSA level was ≥40 ng/ml in 33 patients and < 40 ng/ml in 170 patients. Clinical stage was ≤ T2b in 85 patients, T2c in 19 patients, T3a in 62 patients and ≥ T3b in 37 patients. Almost half of the cohort had ≥ stage T3 disease.

**Table 1:** Patient characteristics

Parameter	All patients		Patient’s with biochemical failure	
	*N*=203	%	*N*=30	%
Age (year)				
Median (range)	75 (56–89)	-	74.5 (56–86)	-
Clinical stage				
T1-T2a	39	19.2	3	10
T2b-c	65	32	10	33.3
T3a	62	30.5	9	30
T3b-T4	37	18.3	8	26.7
Gleason score				
≤ 6	15	7.4	1	3.3
7	45	21.2	8	26.7
8–10	143	70.4	21	70
PSA				
Median (range)	16 (1.4–449)	-	22.7 (1.4–449)	-
<10	71	35	8	26.66
10–20	48	23.6	6	20
20.1–39.9	51	25.1	5	16.66
≥40	33	16.3	11	36.66
>4 cores positive with Gleason 8–10				
≤4	109	53.7	12	40
>4	82	40.4	17	56.7
Unknown	12	5.9	1	3.3
Primary Gleason pattern				
<5	186	91.6	25	83.3
5	17	8.4	5	16.7
NCCN risk group				
High risk	100	49.3	11	36.7
VHR	103	50.7	19	63.3
RT technique				
3D	29	14.3	6	20
IMRT	60	29.6	11	36.7
VMAT	114	56.1	13	43.3
ADT use				
Yes	197	97	28	93.3
No	6	3	2	6.66
Duration ≤ 6 mo	14	6.9	0	0
Duration 6–24 mo	9	4.4	2	6.66
Duration ≥ 24 mo	174	85.7	26	86.66
Prostate radiation dose				
<78 Gy 2 Gy/fx.	5	2.5	1	3.3
78–82 Gy 2 Gy/fx	97	47.8	18	60
73.6 Gy 2.3 Gy/fx	101	49.7	11	36.7
Pelvic lymph node RT				
Yes	201	99	30	100
No	2	1	0	0
46 Gy	88	43.3	16	53.3
54.4 Gy	113	55.7	14	46.7

### NCCN Risk group stratification

The study population included all patients with clinical stage ≥ T3a, or Gleason score ≥8, or PSA > 20 ng/ml. These patients were sorted according to the NCCN definitions of HR and VHR using the original or revised criteria for VHR: (≥ T3B) or (≥T3b or primary Gleason 5 or ≥ 5 cores with Gleason 8–10). Since patients with ≥ 2 HR factors present may be considered as either HR or VHR, and this upstaging is not applied universally, the original and revised NCCN groups were each considered ± upstaging for patients with ≥2 HR factors. The 4 different HR/ VHR groupings created using NCCN criteria were compared.

### Planning and treatment guidelines

All patients received high dose CRT to the prostate and seminal vesicles, pelvic lymph node RT (PLNRT) and ADT. Contouring and planning guidelines evolved over time and guidelines in current use are described below.

Prostate and Seminal Vesicles: The prostate was contoured on axial images from the treatment planning CT scan. The entire seminal vesicles were contoured separately. The prostate and seminal vesicles were combined to create the CTV and then expanded 1 cm in all directions except for 0.7 cm posteriorly to create the PTV. The PTV and CTV were planned to 95 and 98% of the prescribed dose respectively. Three treatment protocols were used for treatment: From 2001 to 2009, 30 patients received 3D CRT to 78Gy-82Gy at 2 Gy/fx; from 2004 to 2011, 72 patients received IMRT to 78Gy-82Gy at 2 Gy/fx, and from 2010 to 2012, 101 patients received VMAT and hypo-fractionation to 73.6Gy at 2.3Gy/fx (80Gy 2gy/eq., σ/β = 1.5). Image guided radiation therapy (IGRT) was introduced into the clinic in 2009 and daily on-line correction was performed daily for all patients [7].

#### PLNRT

Pelvic lymph nodes were identified by contouring and expanding by 7 mm the distal common iliac vessels and external iliac vessels from L5/S1 to the femoral head and symphysis pubis, carving out bowel, bladder and bone. PLNRT was given at 46 Gy at 2Gy/fx. After 2011, PLNRT was given at 54.4 Gy at 1.7 Gy/fx (50Gy 2 Gy/eq, α/β = 1.5). All but 2 patients received PLNRT.

#### Organs at risk

The bladder, rectum from anus to sacral promontory, loops of bowel and femoral heads were contoured. Small bowel dose was limited to 54 Gy with no more than 2 cc receiving 50 Gy. Rectal dose was limited to V75 < 15%, V70 < 25% and V50 < 50%. Bladder dose was V80 < 15%, V75 25%, V65 < 65% and femoral head dose was < 40Gy.

#### ADT

ADT was prescribed for 6 months-3 years. ADT duration: ≤ 6 months *n* = 14 (7%); >6 months- <24 months *n* = 9 (4.4%); ≥24 months *n* = 174 (86%). Six patients (3%) received no ADT and 1 of these did not receive PLNRT.

#### Follow up

Median follow-up for the entire cohort was 50 months (m) (range: 12 m-142 m). Follow-up evaluations following CRT were performed at intervals of 6 m to 1 year. Patients who did not appear for follow-up were contacted telephonically and PSA results were obtained from the electronic medical record.

Biochemical recurrence (BR): When BR was detected, patients underwent diagnostic evaluation with bone scan and CT scans. If the site of recurrence was not identified, patients were offered imaging with choline PET-CT or endorectal MRI (e-MRI), [8]. ADT was not started unless metastatic disease was found. Patients with local, regional, or oligo-metastatic recurrence were offered focal radiation therapy with short term ADT at the discretion of the treating physician.

#### Endpoints

Endpoints used include biochemical disease free survival (b-DFS), (Phoenix definition) [9], distant metastasis free survival (DMFS), prostate cancer specific survival (PCSS) and overall survival (OS). Toxicity and side effects were recorded using CTCAE version 4 [10].

#### Statistics

Statistical analysis was performed using STATA. Continuous and categorical variables were compared using a two-tailed Students t-test or Chi-squared test respectively. The Kaplan-Meier (KM) method was used to calculate probability of survival and toxicity. Cox univariate analysis was conducted using log-rank tests and univariate predictors with a *p*-value <0.2 were further considered using a Cox multivariate proportional hazards model (MVA) to identify predictors of gastrointestinal (GI) or genitourinary (GU) toxicity and survival. *P* values ≤ .05 were considered significant.

## Results

Failure occurred in 30/203(15%) patients. Median time to failure was 30 m (range: 4 m-76 m). Failures were classified as BR only (*n* = 4), local (*n* = 1) or metastatic (*n* = 25). Table 2 lists the sites of failure. In 8 patients who were initially classified as BR, the use of choline PET-CT imaging showed the location and extent of recurrence. Prostate cancer specific mortality was recorded in 4 patients. Eleven deaths were unrelated to prostate cancer.

**Table 2:** Sites of Recurrence

0	173	85.2	
Biochemical failure	4	2	
M1a	3	1.5	2
M1b	16	7.9	4
M1c	6	3	1
Local	1	0.5	1

M1a = non regional lymph nodes, M1b =bones, M1c = visceral

When several sites of recurrence were present, the most advanced category was used

Four different NCCN HR/VHR groupings were evaluated. Patients shifted from the HR group to the VHR group as definitions evolved and additional risk features were included (Table 3). The size of the VHR group increased from 18 to 68% of the patients and HR group decreased from 82 to 32%.

**Table 3:** Patient distribution by NCCN HR and VHR risk groups and 4 year b-DFS, MFS, CSS and OS

	2014		2014+>2 HR		2015		2015+>2 HR	
	HR	VHR	HR	VHR	HR	VHR	HR	VHR
N	166	37	131	72	100	103	65	138
%	82%	18%	64.5%	35.5%	49%	51%	32%	68%
4y bDFS % (95% CI)	89 (82–93)	83 (66–92)	89 (82–93)	84 (73–91)	90 (82–95)	85 (75–91)	92 (80–97)	85 (78–90)
4y MFS % (95% CI)	90 (84–94)	83 (63–93)	92 (84–96)	85 (72–92)	93 (85–97)	85 (75–92)	93 (78–98)	87 (80–92)
4y CSS % (95% CI)	100	92 (70–98)	100	95 (82–99)	100	97 (88–99)	100	98 (91–99)
4y OS % (95% CI)	96 (92–99)	85 (64–94)	98 (93–100)	87 (74–94)	96 (89–99)	92 (83–96)	100	91 (84–96)

HR = High Risk, VHR = Very High Risk, b-DFS = biochemical disease free survival, MFS = metastasis free survival, CSS = cause specific survival, OS = overall survival

NCCN 2014 HR= Stage: T3a, Gleason: 8–10, PSA>20, VHR= T3B, T4

NCCN 2015 HR= Stage: T3a, Gleason: 8–10, PSA>20, T3b-T4, Primary Gl 5, >4 cores with Gl 8–10

KM estimate of 4 year b-DFS for the entire cohort was 87% (95%CI: 82–92%). The 4 year KM survival estimates for b-DFS, CSS and OS were comparable for each of the NCCN subgroups (Table 3) and this was confirmed by Cox regression. On univariate analysis, the NCCN subgroups were not predictive of b-DFS at 4 years. Only DMFS was worse for the VHR group for both pre and post revision NCCN definitions (*p* = .03 and .01 respectively). This difference was not observed if upstaging using ≥2HR factors was applied and this effect did not persevere on MVA. Cox univariate analysis was also significant for: PSA ≥40 *p* = 0.001; clinical stages T2c *p* = .004, T3b *p* = .02 and > 4 cores with Gleason 8–10 *p* < .03.

**Fig. 1:**
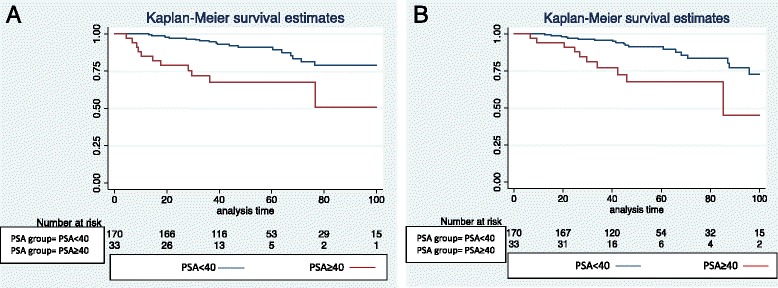
**a** and **b** KM Survival Estimate Stratified by PSA ≥ 40 ng/ml, <40 ng/ml showing Biochemical Disease Free Survival (Fig. 1a) and Metastasis Free Survival (Fig. 1b)

On MVA, PSA ≥ 40 ng/ml was the only significant predictor of b-DFS or DMFS at 4 years with a HR of 3.75 and 3.25, *p* < 0.005 (Table 4). KM estimates for PSA above and below 40 ng/ml are shown in Figs. 1a and b.

**Table 4:** Univariate and Multivariate Survival Analysis

Variable	Univariate analysis		Multivariate analysis	
	*P*-value	HR (95% CI)	*P*-value	HR (95% CI)
Clinical stage				
T1-T2a	-	1.0 (reference)	-	-
T2b-c	0.162	2.51 (0.69–9.16)	-	-
T3a	0.227	2.23 (0.6–8.28)	-	-
T3b-T4	0.045	3.91 (1.02–14.86)	0.138	1.33 (0.91–1.93)
Gleason score				
≤ 6	-	1.0 (reference)	-	-
7	0.223	3.65 (0.45–29.42)	-	-
8–10	0.34	2.66 (0.35–19.87)	-	-
PSA				
<40	-	1.0 (reference)	-	-
≥40	<0.001	3.84 (1.82–8.080	0.001	3.75 (1.76–7.97)
>4 cores positive with Gleason 8–10				
≤4	-	1.0 (reference)	-	-
>4	0.032	2.27 (1.07–4.8)	0.23	1.41 (0.8–2.51)
Unknown	0.635	0.6 (0.07–4.7)	-	-
Primary Gleason pattern				
<5	-	1.0 (reference)	-	-
5	0.124	2.12 (0.81–5.55)	-	-
NCCN risk				
High risk	-	1.0 (reference)	-	-
VHR	0.07	1.99 (0.94–4.23)	-	-
RT technique				
3D	-	1.0 (reference)	-	-
IMRT	0.392	1.57 (0.55–4.41)	-	-
VMAT	0.376	1.67 (0.53–5.22)	-	-

Treatment was well tolerated with significant late ≥ grade 3 GU toxicity of 10% which was predominantly due to reports of nocturia more than 5 times per night. Late ≥ grade 3 GI toxicity of 3.5% was due to rectal bleeding (Table 5).

**Table 5:** Toxicity Profile

	GI toxicity		GU toxicity	
	Acute (%)	Late (%)	Acute (%)	Late (%)
Grade 0	140 (69%)	166 (82%)	53 (26%)	117 (58%)
Grade 1	58 (29%)	17 (8%)	112 (55%)	31 (15%)
Grade 2	3 (1.5%)	13 (6.4%)	32 (16%)	34 (17%)
Grade 3	2 (1%)	7 (3.5%)	6 (3%)	21 (10%)
Grade 4	-	-	-	-

## Discussion

This study supports the assertion that patients with both HR and VHR prostate cancer treated with high dose CRT, PLNRT and ADT have favorable outcomes with low toxicity. The use of dose escalation, CRT, PLNRT, image guidance and ADT have all been associated with improved outcomes or reduced toxicity [11–15] and the results of this study are consistent with favorable outcomes reported from studies that used CRT with ADT to treat patients with HR disease [16]. While our results are encouraging, these findings must be interpreted cautiously since longer follow-up time is needed to verify our findings.

Dissimilarities in risk factors and co-morbidities of HR patients treated with RP or CRT make comparison of outcomes between surgical and radiation treatments unreliable. Series reporting treatment outcomes for CRT with HR disease include many patients with advanced disease or comorbidities that would exclude consideration for RP. Despite the inclusion of patients with adverse risk factors, our results compare well to outcomes reported for HR patients treated with RP [2, 17, 18]. Furthermore, we report low rates of acute and late GI and GU toxicity that are consistent with other CRT series [19]. In contrast, HR patients treated with RP often require adjuvant or salvage RT which is associated with increased toxicity when compared to treatment with RP or RT alone [20, 21].

The 2015 revisions to the VHR subgroup were based on the findings of Sundi et al. who reviewed prognostic factors and outcomes from a surgical series of 753 men with HR prostate cancer to create risk factor groupings predictive for metastatic disease and prostate cancer specific mortality [4]. Based on Sundi’s findings, the revised 2015 NCCN guidelines added two additional criteria for inclusion of HR patients into the VHR subgroup [1, 4]. When subdividing our CRT series into HR/VHR subgroupings, we observed that stage migration was substantial. The percentage of patients considered VHR increased from 18 to 62% as the number of criteria considered for inclusion in the VHR subgroup increased. We suggest that redistribution of patients into the VHR group may improve the reliability of comparisons of HR patients treated primarily with surgery versus those treated with radiation.

We found that patients treated with high dose CRT and ADT did well irrespective of current HR/VHR classification and that PSA ≥40 ng/ml was the best discriminator of poor outcomes. In contrast to our findings, Narang et al. showed that when evaluating patients with HR disease who were treated over a 15 year interval from 1993 to 2006, the revised 2015 NCCN HR/VHR subgrouping were predictive for worse outcomes in the VHR subgroup [6]. Although the long follow-up interval is an important strength of Narang’s study, the median follow-up time of our cohort is 50 m (range: 12 m-142 m) and the median time to BF in our reports are similar at 34 m and 30 m. The difference in our findings may be better explained by the differences in treatment received by our respective cohorts. Narang reported the use of a diverse assortment of radiation techniques with a mean dose of 70.2 Gy (range: 64.8–75.6 Gy), and differing ADT protocols. Narang was unable to demonstrate improvement in failure endpoints with dose escalation over 72Gy although use of neo-adjuvant ADT was associated with reduced BF and DMFS. In comparison, the patients in our series received uniform CRT with a minimum dose of 78 Gy (range: 78–82 Gy), PLNRT, and ADT that is reflective of current practice. Increased radiation dose and use of ADT have been shown to be associated with improved b-DFS and DMFS [11, 22, 23]. Pollack et al. in a randomized trial showed that doses <78 Gy versus ≥ 78 Gy were associated with improved b-DFS and DMFS and Denham et al. reported that both dose escalation and increased duration of ADT reduced local progression and BF [22, 23].

The only risk factor predictive for reduced b-DFS or DMFS on MVA in our series was PSA ≥40 ng/ml. This finding is consistent with other studies showing elevated PSA at time of diagnosis to be highly predictive for metastatic disease following either RT or RP [24–27]. These findings suggest that future modifications to the NCCN guidelines consider PSA level as a criteria for inclusion into the VHR group. We caution that elevated PSA should not be used as an exclusion criteria for definitive treatment since many patients with elevated PSA may benefit from definitive therapy [24].

Patterns of failure analysis shows that distant failure was dominant and isolated initial failure within the lymph nodes, prostate or as BF alone was uncommon. The use of functional imaging has allowed us to identify sites of distant failure early which decreased the number of patients considered LR or BF alone. In contrast to our findings, several studies which used bone scan, CT and prostate biopsy to evaluate patients with BF have reported that the prostate is the most common first site of failure following RT for HR patients [28, 29]. Although prostate biopsy results were not available in our series, choline PET-CT and endorectal-MRI imaging in patients with BF allowed for early detection of metastatic disease in most patients. These findings support the use of CRT with ADT to treat patients with HR disease and suggest that further intensification of local therapy will provide little benefit for HR patients and may only add morbidity. [2, 3, 17, 30, 31].

The NCCN guidelines were revised in 2014 to include choline PET-CT imaging of patients with BF for consideration of focal salvage therapy [32]. Early adaptation of functional imaging with choline PET-CT allowed us to offer patients with local failure or oligo-metastatic disease salvage treatment using targeted radiation therapy and avoid early administration of ADT [8]. Extending the use of functional imaging agents for use during initial staging may further improve outcomes with RP and CRT by identifying and excluding patients with early metastatic disease from receiving definitive therapy.

The absence of a central pathology review is an important study limitation. Since patients were referred from several different institutions, variation between pathologists in assigning Gleason grades to the biopsy specimens may have affected the classification of our patients into HR and VHR subgroups. Although several different CRT treatment techniques were used during the study period and moderate hypo-fractionation and IGRT were instituted only after 2009, univariate and multivariate analysis were unable to demonstrate differences in outcome based on treatment technique. Although most BFs occur within 5 years of treatment [33], longer duration of follow-up is needed to verify our findings.

## Conclusions

Prostate cancer patients with HR and VHR disease achieve excellent LC and DMFS with low toxicity when treated with dose escalated CRT, PLNRT and ADT. Reclassification of HR patients into HR/VHR subgroups using original or revised NCCN criteria had no impact on treatment outcomes. Only PSA ≥40 ng/ml was associated with poor prognosis. The use of functional imaging to evaluate BF showed that distant failure was dominant and LR in the prostate rare, challenging the notion that intensification of local therapy will further improve outcomes. Further study and longer follow-up is required to validate these findings.

## Abbreviations

ADT: Androgen Deprivation Therapy
B-DFS: Biochemical Disease Free Survival
BF: Biochemical Failure
BR: Biochemical Recurrence
CI: Confidence Interval
CRT: Conformal Radiation Therapy
CTV: Clinical Target Volume
GI: Gastrointestinal
GU: Genitourinary
HR: Hazard Ratio
HR: High Risk
IGRT: Image Guided Radiation Therapy
IMRT: Intensity Modulated Radiation Therapy
KM: Kaplan Meier
LR: Local Recurrence
M: Months
MFS: Metastasis Free Survival
MVA: Multivariate Analysis
NCCN: National Comprehensive Cancer Network
OS: Overall Survival
PCSS: Prostate Cancer Specific Survival
PLNRT: Pelvic Lymph Node Radiation Therapy
PSA: Prostate Serum Antigen
PTV: Planning Target Volume
RP: Radical Prostatectomy
RT: Radiation Therapy
VHR: Very High Risk
VMAT: Volume Modulated Radiation Therapy
